# Control of Spontaneous HPV16 E6/E7 Expressing Oral Cancer in HLA-A2 (AAD) Transgenic Mice with Therapeutic HPV DNA Vaccine

**DOI:** 10.1186/s12929-021-00759-x

**Published:** 2021-09-13

**Authors:** Ssu-Hsueh Tseng, Li Liu, Shiwen Peng, Jinhwi Kim, Louise Ferrall, Chien-Fu Hung, T. -C. Wu

**Affiliations:** 1 Department of Pathology, Johns Hopkins University, CRB II, 1550 Orleans St, Baltimore, MD 21287 USA; 2 Department of Oncology, Johns Hopkins University, CRB II, 1550 Orleans St, Baltimore, MD 21287 USA; 3 Department of Obstetrics and Gynecology, CRB II, Johns Hopkins University, 1550 Orleans St, Baltimore, MD 21287 USA; 4 Department of Molecular Microbiology and Immunology, CRB II, Johns Hopkins University, 1550 Orleans St, Baltimore, MD 21287 USA; 5 Department of Obstetrics and Gynecology, Uijeongbu St. Mary’s Hospital, College of Medicine, The Catholic University of Korea, 271, Cheonbo-Ro, Uijeongbu, Gyeonggi-do 11765 Republic of Korea; 6 Departments of Pathology, Oncology, and Obstetrics and Gynecology, The Johns Hopkins Medical Institutions, CRB II Room 307, 1550 Orleans St, Baltimore, MD 21231 USA; 7 Departments of Pathology, Oncology, Obstetrics and Gynecology, and Molecular Microbiology and Immunology, The Johns Hopkins Medical Institutions, CRB II Room 309, 1550 Orleans St, Baltimore, MD 21231 USA

**Keywords:** HPV, DNA vaccine, Preclinical model, HPV16, Transgenic mice, HLA-A2

## Abstract

**Background:**

Human Papillomavirus type 16 (HPV16) has been associated with a subset of head and neck cancers. Two HPV encoded oncogenic proteins, E6 and E7, are important for the malignant progression of HPV-associated cancers. A spontaneous HPV16 E6/E7-expressing oral tumor model in human HLA-A2 (AAD) transgenic mice will be important for the development of therapeutic HPV vaccines for the control of HPV-associated head and neck cancers.

**Methods:**

In the current studies, we characterized the HLA-A2 restricted HPV16 E7-specific CD8 + T cell mediated immune responses in the HLA-A2 (AAD) transgenic mice using a therapeutic naked DNA vaccine encoding calreticulin (CRT) linked to a mutated E7(N53S). We also employed oncogenic DNA plasmids that encoded HPV16E6/E7/Luc, NRas^G12V^, and sleeping beauty transposase for the transfection into the submucosal of oral cavity of the transgenic mice with electroporation to create a spontaneous oral tumor. Furthermore, we characterized the therapeutic antitumor effects of CRT/E7(N53S) DNA vaccine using the spontaneous HPV16 E6/E7-expressing oral tumor model in HLA-A2 (AAD) transgenic mice.

**Results:**

We found that CRT/E7(N53S) DNA vaccine primarily generated human HPV16 E7 peptide (aa11-20) specific CD8 + T cells, as compared to the wild-type CRT/E7 vaccine, which primarily generated murine H-2D^b^ restricted E7 peptide (aa49-57) specific CD8 + T cell responses. We also observed transfection of the oncogenic DNA plasmids with electroporation generated spontaneous oral tumor in all of the injected mice. Additionally, treatment with CRT/E7(N53S) DNA vaccine intramuscularly followed by electroporation resulted in significant antitumor effects against the spontaneous HPV16 E6/E7-expressing oral tumors in HLA-A2 (AAD) transgenic mice.

**Conclusions:**

Taken together, the data indicated that the combination of HPV16 E6/E7-expressing DNA, NRas^G12V^ DNA and DNA encoding sleeping beauty transposase is able to generate spontaneous oral tumor in HLA-A2 (AAD) transgenic mice, which can be successfully controlled by treatment with CRT/E7(N53S) DNA vaccine. The translational potential of our studies are discussed.

**Supplementary Information:**

The online version contains supplementary material available at 10.1186/s12929-021-00759-x.

## Background

Human Papillomavirus (HPV) is a major cause of several human cancers, including cervical, penile, vaginal, vulvar, anogenital, and a subset of head and neck (HN) cancers. High-risk HPV types (hrHPV) HPV16 and 18 dominantly incite HPV-associated cancers. Since the 1980s, the incidence of HPV-associated HN cancer has risen significantly in the United States and other developed nations [1]. HPV now accounts for about 65–70% of cases of oropharyngeal cancer diagnosed in the U.S. each year, of which about 90% are HPV16 associated [2–5]. HPV oncogenes E6 and E7 are the major oncogenic drivers of HPV-associated cancers and tumorigenesis [6, 7]. Immunotherapy treatment works by priming the host immune system to target tumor and malignant tissues. HPV encoded oncogenic proteins E6 and E7 represent potentially ideal targets for the development of immunotherapy against HPV-associated malignancies because they are foreign antigens that are not subject to self-tolerance and they are expressed only in infected or cancerous cells but not in normal cells. Currently, although HPV-associated HN cancer patients generally respond better to standard-of-care chemoradiation compared to non-HPV-associated HN cancer patients [8, 9], patients experience significant treatment morbidity. Additionally, patients with recurrent or metastatic HPV-associated HN cancer can have dismal prognosis. Therefore, development of novel treatments that can limit morbidity and effectively treat recurrent/metastatic HN cancer is desperately needed.

Therapeutic DNA vaccines are a potentially promising immunotherapeutic strategy for the treatment of HPV-associated cancers, including HN cancers due to its remarkable features such as safety, stability and easy for preparation. To date, several therapeutic HPV DNA vaccines targeting hrHPV E6 and E7 proteins have been developed (for review see [10, 11]). However, one of the major drawbacks to DNA vaccines is that they can be poorly immunogenic and fail to incite a potent antitumor immune response due to poor transfection efficiency following injection in vivo. Thus, strategies to enhance immunogenicity are typically required to elicit observable antitumor effect generated by naked DNA vaccine (For review see [10, 12]). Among these strategies, DNA vaccines encoding calrecticulin (CRT) linked to antigens have been shown to generated potent antigen-specific immune responses and antitumor effects [13–18]. CRT is a calcium-binding protein located on the endoplasmic reticulum and is one of the heat shock proteins. Previous research suggests that CRT linked to E7 antigen in a context of DNA vaccine has been shown to enhance major histocompatibility complex (MHC) class I presentation of HPV16 E7 antigen to activate E7-specific CD8 + T cells [13, 15]. In addition, the CRT/E7 vaccine has previously been shown to generate potent therapeutic antitumor effects against an E7-expressing tumor in a preclinical mouse model [13, 15].

In order to test the efficacy of therapeutic DNA vaccine, it is important to have a suitable preclinical model. A relevant preclinical HPV-associated oral tumor model should spontaneously develop in oral tissue, constitutively express HPV E6/E7, have an intact immune system that is amenable to immunotherapeutic treatment, and tumor formation should mirror clinical progression from precancerous to cancerous states [19]. Based on these features, we have previously successfully developed a spontaneous HPV16 E6/E7 expressing oral tumor model in wild-type C57BL/6 mice [19]. In order to ensure the preclinical model is relevant for human clinical translation, it will be important to build a preclinical tumor model using human MHC class I transgenic mice. Among different human MHC class I molecules, HLA-A2 is one of the most common human MHC-I haplotypes in Americans [20]. As such, we used HLA-A2 (AAD) transgenic mice for the development of our tumor model. HLA-A2 (AAD) transgenic mice express chimeric MHC I molecules, comprising of human HLA class I molecules expressing alpha-one, alpha-two domain of HLA-A0201 from human and alpha three transmembrane cytoplasmic domain of H-2D^b^ derived from the mouse [21]. This transgenic mouse is commonly used to model human T cell immune responses to HLA-A2 presented antigens.

Here, we described the development of a spontaneous HPV16 E6/E7-expressing oral tumor model using HLA-A2 (AAD) transgenic mice. However, the AAD transgenic strain also expresses murine H-2D^b^ MHC class I molecule. Because it has been shown that the presence of murine H-2D^b^ restricted CTL epitope (aa49-57) would suppress the presentation of human HLA-A2 restricted CTL epitope (aa11-20) [22], we reasoned that a mutation in the E7 (aa49-57) CTL epitope in the E7 gene of CRT/E7 DNA vaccine would eliminate murine H-2D^b^ restricted E7 peptide (aa 49–57)-specific CD8 + T cell mediated immune responses and result in enhanced HLA-A2 restricted HPV16 E7 peptide (aa11-20) specific CD8 + T cell mediated immune responses in vaccinated HLA-A2 (AAD) transgenic mice. It has been shown that a mutation at location 53 from N to S can abolish the presentation of H-2D^b^ restricted E7 (aa49-57) peptide specific CTL epitope [23]. We therefore created a mutated E7 gene by replacing the amino acid of asparagine (N) with amino acid of serine (S) at location 53 (N53S) and used CRT/E7(N53S) DNA vaccine to characterize the H-2D^b^ -restricted E7 peptide (aa49–57)-specific CD8 + T-cell immune response as well as the HLA-A2 restricted E7 peptide (aa11-20)-specific CD8 + T cell immune response in HLA-A2 (AAD) transgenic mice. We also employed the CRT/E7(N53S) DNA vaccine to determine the antitumor effects against the HPV16E6E7-expressing spontaneous oral tumors in the HLA-A2 (AAD) transgenic mouse model. We observed that vaccination with CRT/E7(N53S) DNA vaccine generated potent human HLA-A2 restricted HPV16 E7 specific CD8 + T cells mediated immune responses and therapeutic antitumor effects against the spontaneous oral tumors in the HLA-A2 (AAD) transgenic mouse model. Our work has significant translational potential.

## Methods

### Mice

HLA-A2 (AAD) transgenic mice were kindly provided by Dr Victor Engelhard at the University of Virginia [21]. HLA-A2 (AAD) transgenic mice contain chimeric HLA class I molecules expressing alpha-one, alpha-two domain of human HLA-A2 and alpha three transmembrane cytoplasmic domain of H-2D^d^ derived from mice. HLA-A2 (AAD) transgenic mice were then bred at the animal facility. Johns Hopkins University School of Medicine Animal Facility (Baltimore, MD) is responsible for maintaining all mice under specific pathogen-free conditions. Johns Hopkins Institutional Animal Care and Use Committee approved all procedures which were performed as stated by protocols and all laboratory animals are under proper care. Female mice aged six to eight weeks were used for this study.

### Plasmids

The construction of pT/Caggs-NRasV12 plasmid has been described previously [24]. The construction of pCMV(CAT)T7-SB100 plasmid has also been described previously [25]. The plasmids were purchased from Addgene (plasmid #20205, Addgene #34879).

The generation of Pkt2-Luc-T2a-E7-T2a-E6 has been described previously [19].

The generation of pcDNA-3-CRT/E7 (CRT/E7) has been previously described [13]. HPV16 E7 one point N53S mutation reduces stabilization of H-2D^b^ molecules and eliminates immunogenicity of E7, which has been previously described [23]. To generate pcDNA3-CRT-E7(N53S), E7(N53S) was amplified by PCR using pcDNA3-E7 [13] as a template and the following a set of primers, 5′-TTTGAATTCATGCATGGAGATACACCTAC-3′, 5′-TTTAAGCTTTTATGGTTTCTGAGAACAGAT-3′, 5′-AGAACCGGACAGAGCCCATTACAGTATTGTAACCTTTTGTTGCAAGTG-3′ and 5′-CACTTGCAACAAAAGGTTACAATACTGTAATGGGCTCTGTCCGGTTCT-3′ and cloned into the EcoRI/HindIII sites of pcDNA3-CRT [13] to generate CRT/E7(N53S).

### Immunization of the DNA vaccine

For the characterization of E7 antigen-specific CD8 + T cell mediated immune responses, HLA-A2 (AAD) transgenic mice (5 per group) were immunized with either CRT/E7 or CRT/E7(N53S) DNA vaccine. Female HLA-A2 (AAD) transgenic mice were vaccinated with 10 μg/mouse of either CRT/E7 or CRT/E7(N53S) DNA vaccine on day 0 through intramuscular injection followed by electroporation. Vaccines were injected once a week for two consecutive weeks. One week after final vaccination, PBMCs or splenocytes from vaccinated mice were isolated and analyzed for HPV16 E7 antigen-specific immune response.

### Flow cytometry analysis

To detect HPV16 E7-specific CD8^+^ T cell responses by IFN-γ intracellular staining, splenocytes from vaccinated HLA-A2 (AAD) transgenic mice were harvested 12 days after last vaccination with CRT/E7 or CRT/E7(N53S) and stimulated with either HPV16 E7 overlapping peptides (15mer with 10aa overlap) (5 μg/ml) or HPV16 E7 short peptides (HPV16 E7aa11-20, E7aa49-57, E7aa82-90 and E7aa86-93 peptides) [26] (1 µg/ml) at the presence of GolgiPlug (BD Pharmingen, San Diego, CA) at 37 °C overnight. The stimulated splenocytes were stained PE anti-mouse CD8a (BD Biosciences, Cat. #561,095) followed by fixation and permeabilization using the Cytofix/Cytoperm kit according to the manufacturer’s instruction (eBioscience, San Diego, CA). Intracellular IFN-γ was stained with FITC anti-mouse IFN-γ (Biolegend, Cat. # 505806). The cells were acquired with FACSCalibur flow cytometer and data were analyzed with CellQuest Pro software.

For the HPV16 E7 tetramer staining, PBMCs were collected from submandibular vein of mice cheeks and collected in EDTA-containing eppendorf tubes. Red blood cell lysis buffer, ACK lysing buffer (Quality Biological, Gaithersburg, MD, USA), was added to the blood sample to lysis red blood cells, then washed with a FACS washing buffer containing phosphate-buffered saline (PBS) with 0.5% bovine serum albumin (BSA). Zombie Aqua live/dead (BioLegend Inc., Cat. #423102) was used for dead cell exclusion. Following Fc blocking, cells were stained with FITC-conjugated anti-mouse CD8 (Biolegend, Cat. # 100712), PE-conjugated H-2D^b^ tetramer loaded with HPV16-E7 aa49–57 peptide or PE-conjugated HLA-A2 tetramer loaded with HPV16-E7 aa11–20 peptide (provided by NIH Tetramer Core Facility). The stained cells were washed with FASC washing buffer, and followed by FACS analysis. To gate the E7-specific T cells, single cells were gated by side scatter wide and side scatter area. Subsequently, live cells were gated by side scatter area and live/dead negative population. Live lymphocytes were gated by side scatter area and forward scatter area. Next, E7-specific CD8 + T cells were gated by CD8 and E7-tetramer double positive cells. The PBMCs from unvaccinated mice were used to determine the tetramer-negative cells.

In order to characterize the tumor microenvironment, the isolated Tumor Infiltrating Lymphocytes (TILs) were stained with Alexa Fluor® 488 anti-mouse Ly-6G/Ly-6C (Gr-1)( BioLegend, Inc., Cat. # 108417), PE/Cyanine7 anti-mouse CD3 (BioLegend, Inc, Cat. # 100219), Pacific Blue™ anti-mouse CD45 (BioLegend, Inc., Cat. # 103126), APC anti-mouse CD11b (eBioscience™, Cat. # 17–0112-83), Brilliant Violet 650™ anti-mouse CD8a (BioLegend, Inc., Cat. # 100742), Brilliant Violet 785™ anti-mouse CD4 (BioLegend, Inc, Cat. # 100552), and PE-conjugated HLA-A2 tetramer loaded with HPV16-E7 aa11–20 peptide. To gate the TILs, single cells were gated by side scatter wide and side scatter area. Live CD45 positive cells were gated by CD45 positive and live/dead negative population. Tumor- Infiltrating CD4 + T cells were gated by CD3 and CD4 double positive population. Tumor- Infiltrating CD8 + T cells were gated by CD3 and CD8 double positive population. Myeloid-derived suppressor cells (MDSCs) were gated by Gr1 and CD11b double positive population. FACS analysis was performed using CytoFLEX S (Beckman) and data were analyzed by FlowJo software.

### Generation of spontaneous oral tumor model

Six to 8 week old female HLA-A2 (AAD) transgenic mice were used for the development of the oral tumor model. Mice were immunocompromised through intraperitoneal injection of monoclonal anti-CD3 (Clone: 17A2; Catalog #BE0002, Bio X Cell) for three continuous days (150 µg/mice). On the last day of injection, plasmids encoding mutant NRas^G12V^, SB100, HPV16-E6/E7 and luciferase were submucosally injected into the oral area of mice (10 µg/plasmid, 30 µl/injection) followed by electroporation (8 pulses, 72v for 20 ms/pulse, 20 ms intervals between each pulse). Tumor growth was followed by inspection and luminescent imaging.

### Bioluminescence imaging:

To monitor the tumor growth, IVIS spectrum bioluminescence imaging series 2000 (PerkinElmer) was used. Mice were anesthetized by isoflurane in the image room. Ten minutes after mice receive intraperitoneal injection (IP) with D-luciferin (GoldBio) substrate, bioluminescence imaging for luciferase expression was administered on a cryogenically cooled IVIS system using Living Image acquisition and analysis software (Xenogen). The tumor region was quantified as photon counts using Xenogeny analysis software in Living image 2.5 system. The growth of tumor was observed once a week for four weeks through luminescence imaging.

### Histology and immunohistochemistry staining

Oral tumors were surgically removed on day 21 and put into 10% neutral buffered formalin solution for adequate fixation for at least 48 h in room temperature after euthanizing the spontaneous HPV16 E6/E7-expression oral tumor-bearing mice. The tumor tissues were formalin fixed and paraffin embedded. Johns Hopkins University Oncology Tissue Services were responsible for performing hematoxylin and eosin (H&E) staining of tissue sections. A board-certified gynecologic pathologist (Dr. T.-C. Wu) of the Pathology Department in the Johns Hopkins University School of Medicine assisted in reviewing the histology slides.

### Anti-tumor DNA vaccine treatment

HLA-A2 (AAD) transgenic mice (n = 5) underwent CD3 + T cell depletion through intraperitoneal injection of monoclonal anti-CD3 (Clone: 17A2; Catalog #BE0002, Bio X Cell) for three continuous days (150 µg/mice). Following the final injection on day 3, plasmids encoding mutant NRas^G12V^, SB100, HPV16-E6/E7 and luciferase were submucosally injected into the oral area of mice (10 µg/plasmid, 30 µl/injection), followed by electroporation. One week after the injection of oncogenic plasmid, one group of the tumor-bearing HLA-A2 (AAD) transgenic mice was treated with CRT/E7(N53S) DNA vaccine (10 µg/plasmid, 30 µl/injection) via intramuscular injection (IM) into the shaved leg region, followed by electroporation. A second group was treated with empty pcDNA3 plasmid vector (10 µg/plasmid, 30 µl/injection) via IM injection into the shaved leg region, followed by electroporation. Mice received DNA treatment twice a week for consecutive two weeks (day 10, 13, 16, 19). Tumor growth was then followed by luminescent imaging. When the tumor reached over 7 mm in diameter, mice were euthanized.

### Tumor-infiltrating lymphocytes isolation

After the last vaccine injection, splenocytes and tumor tissue of mouse were obtained through surgical removal on day 21 and cutting into 2 mm pieces digested with serum-free RPMI-1640 medium containing collagenase I (0.05 mg/mL; Sigma-Aldrich, Catalog #C9891), collagenase IV (0.05 mg/mL; Sigma-Aldrich, Catalog #C5138), hyaluronidase IV (0.025 mg/mL; Sigma-Aldrich, Catalog #H3506), DNase I (0.25 mg/mL; Sigma-Aldrich, Catalog #DN25-g), and penicillin (100 U/mL) and streptomycin (100 μg/mL) (Thermo Fisher, Catalog #15140122). Processed tumor pieces were incubated at 37 °C with periodic agitation. To remove undigested tissue fragments, the tumor solution was filtered through a 70 μm nylon filter mesh and the solution was washed with FACS buffer.

### Statistical analysis

The statistical analysis was performed using GraphPad Prism V.9 software and data were interpreted as means with standard deviation. Kaplan–Meier survival plots are used to estimate the survival percentage and tumor-free rate. Long rank tests were used to compare the survival time between treatment groups. Comparison between individual data points were used to analyze in the student’s t- test and p value smaller than 0.05 is considered statistically significant. * =  < 0.05, ** =  < 0.01,*** =  < 0.001, ns = not significant.

## Results

### Vaccination of HLA-A2 (AAD) transgenic mice with CRT/E7 DNA vaccine generated potent murine H-2D^b^ restricted HPV16 E7 peptide (aa49-57) -specific CD8 + T cell mediated immune responses

**Fig. 1:**
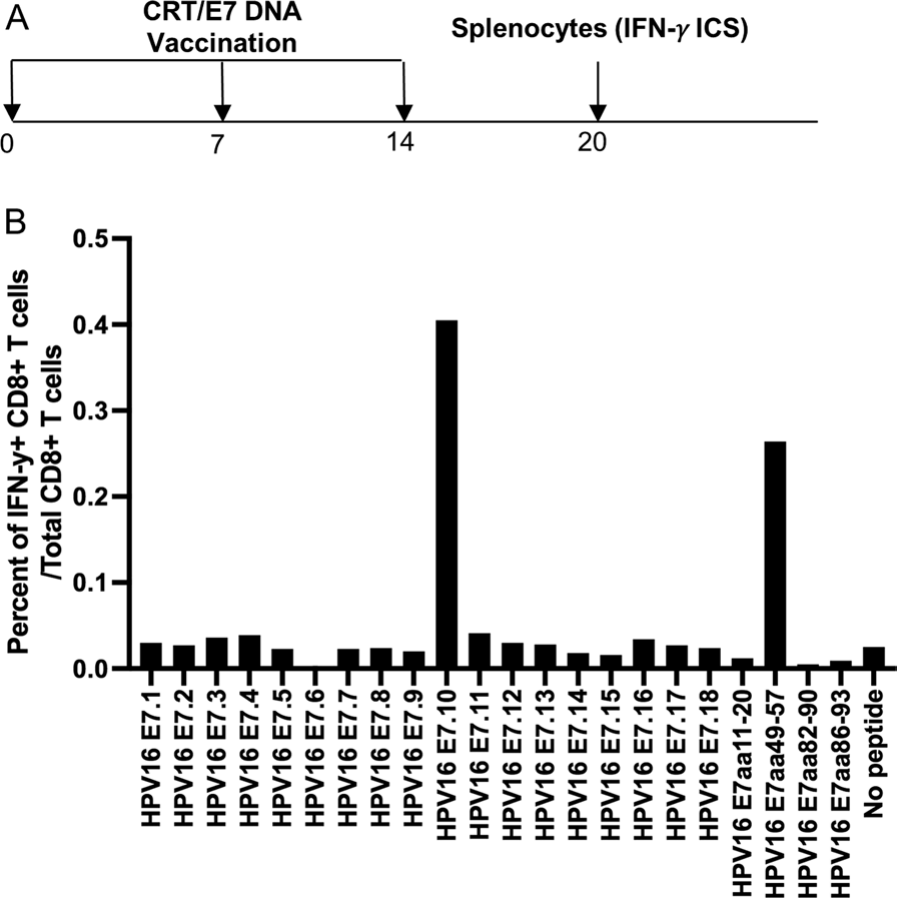
Characterization of HPV16 E7-specific CD8 + T cell mediated immune response in HLA-A2 (AAD) transgenic mice vaccinated with CRT/E7 DNA vaccine.** A** Schema of the experimental regimen. Briefly, female HLA-A2 (AAD) transgenic mice (5 per group) were vaccinated with 10 μg/mouse of CRT/E7 DNA vaccine on day 0 through intramuscular injection followed by electroporation. One week later the mice were boosted twice with the same dose and regime at one week intervals. 6 days after the final booster, the splenocytes from the mice were prepared and stimulated with HPV16 E7 overlapping peptides that span the full-length of the HPV16 E7 protein, HPV16 E7aa11-20, E7aa49-57, E7aa82-90 or E7aa86-93 peptides. The cells were then stained with PE-conjugated anti-mouse CD8a. After permeabilization and fixation, the cells were stained with FITC-conjugated anti-mouse IFN-γ. The stained cells were analyzed by flow cytometry. **B** Bar graph to summarize the percent of activated (IFN-γ +) HPV16 E7-specific CD8 + T cells out of total CD8 + T cells in response to various peptides in HLA-A2 (AAD) transgenic mice after vaccination with CRT/E7 DNA

HLA-A*0201/D^d^ (AAD) transgenic mice express an interspecies hybrid class I MHC gene, AAD, which contains the alpha-1 and alpha-2 domains of the human *HLA-A2.1* gene and the alpha-3 transmembrane and cytoplasmic domains of the mouse *H-2Dd* gene/allele, under the direction of the human *HLA-A2.1* promoter [21]. This transgenic strain allows us to model human T cell immune responses to HLA-A2 presented antigens and may be useful in testing of vaccines for infectious diseases or cancer therapy. However, the AAD transgenic strain also expresses murine H-2D^b^ MHC class I molecule. The AAD transgenic mice were vaccinated with 10 µg/mouse of CRT/E7 DNA vaccine through intramuscular injection followed by electroporation. The mice were boosted twice with the same dose and regimen at one-week intervals (Fig. 1A). Six days after the last vaccination, the splenocytes from the vaccinated mice were harvested and stimulated with HPV16 E7 overlapping peptides that span the full length of the HPV16 E7 protein, HPV16 E7aa11-20, E7aa49-57, E7aa82-90 and E7aa86-93 peptides. The cells were characterized for IFN-γ + CD8 + T cells by intracellular cytokine staining followed by flow cytometry analysis. As shown in Fig. 1B, the AAD mice vaccinated with CRT/E7 predominantly mounted a murine H-2 D^b^ restricted HPV16 E7 peptide (aa49-57) specific CD8 + T cell response. Our data indicated that the presence of murine H-2 D^b^ restricted E7-specific CTL epitope (aa49-57) in the E7 gene of CRT/E7 DNA suppresses the presentation of HLA-A2 restricted E7-specific CTL epitopes.

### Vaccination of HLA-A2 (AAD) transgenic mice with CRT/E7(N53S) DNA vaccine generated potent HLA-A2 restricted HPV16 E7 peptide (aa11-20) specific CD8 + T cell mediated immune responses

**Fig. 2:**
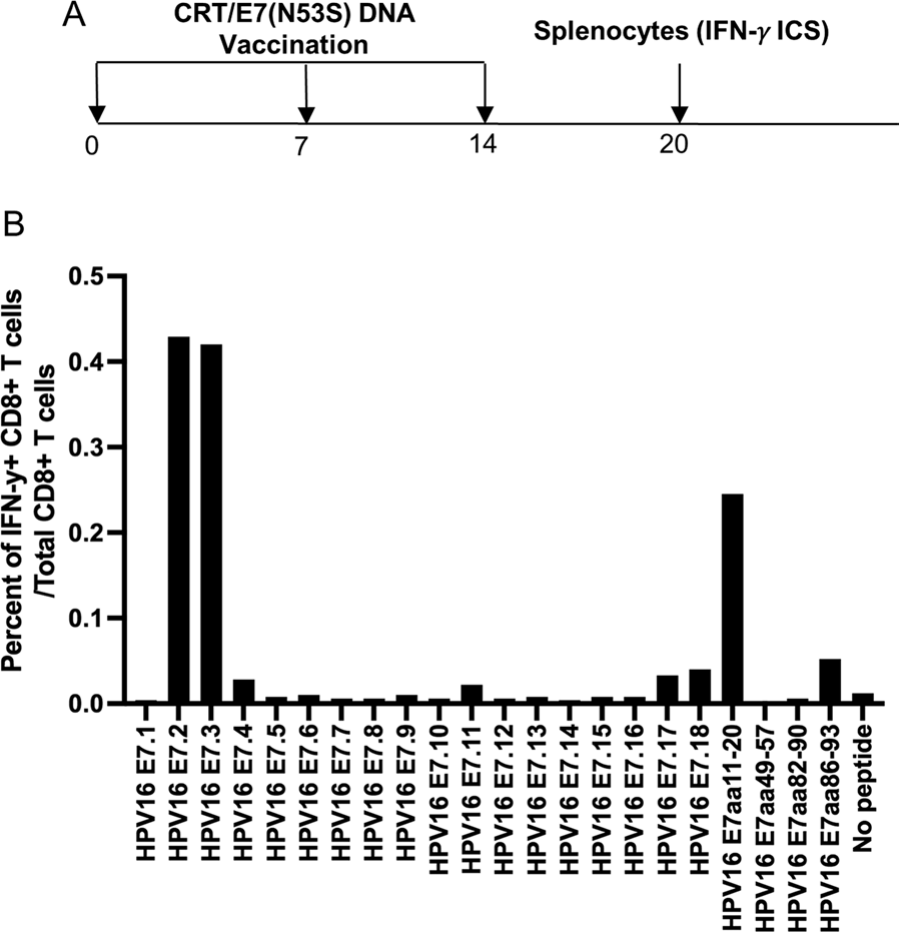
Characterization of HPV16 E7-specific CD8 + T cell mediated immune response in HLA-A2 (AAD) transgenic mice vaccinated with CRT/E7(N53S) DNA vaccine.** A** Schema of the experimental regimen. Briefly, female HLA-A2 (AAD) transgenic mice (5 per group) were vaccinated with 10 μg/mouse of CRT/E7(N53S) DNA vaccine on day 0 through intramuscular injection followed by electroporation. One week later the mice were boosted twice with the same dose and regime at one week intervals. 6 days after the final booster, the splenocytes from the mice were prepared and stimulated with HPV16 E7 overlapping peptides that span the full-length of the HPV16 E7 protein, HPV16 E7aa11-20, E7aa49-57, E7aa82-90 or E7aa86-93 peptides. The cells were then stained with PE-conjugated anti-mouse CD8a. After permeabilization and fixation, the cells were stained with FITC-conjugated anti-mouse IFN-γ. The stained cells were analyzed by flow cytometry. **B** Bar graph to summarize the percent of activated (IFN-γ +) of HPV16 E7-specific CD8 + T cells out of the total CD8 + T cells in response to various peptide stimulation in HLA-A2 (AAD) transgenic mice after vaccination with CRT/E7(N53S) DNA

In order eliminate the concern that the presence of immunodominant murine H-2D^b^ restricted E7-specific CTL epitope (aa49-57) in the E7 gene suppresses the presentation of HLA-A2 restricted E7-specific cytotoxic CTL epitopes in HLA-A2 (AAD) transgenic mice, we have created a new DNA construct encoding CRT linked to mutated E7 gene (CRT/E7(N53S)). We generated E7(N53S) by substituting the amino acid 53 from N to S (RAHYNIVTF to RAHYSIVTF). We vaccinated the HLA-A2 (AAD) transgenic mice with the new CRT/E7(N53S) DNA construct as described in Fig. 1 (Fig. 2A). As shown in Fig. 2B, the alternation of one amino acid at location 53 from N to S completely eliminated H-2D^b^ restricted E7-peptide (aa49-57) CD8 + T cell mediated immune responses in vaccinated mice. Instead, we have observed the increase of HLA-A2 restricted E7-peptide (aa11-20) specific CD8 + T cell mediated immune responses. Overlapping peptides HPV16 E7.2 and E7.3 also increased after vaccination with CRT/E7(N53S), since both of these peptides include the HLA-A2 restricted E7-peptidespecific CTL epitope (aa11-20). Taken together, our data suggest that the mutation at murine H-2D^b^ restricted E7-specific CTL epitope (aa49-57) reduces presentation of the murine H-2D^b^ restricted E7-specific CTL epitope and allows the presentation of human HLA-A2 restricted E7-specific CTL epitope (aa11-20) in HLA-A2 (AAD) transgenic mice.

### Confirmation that CRT/E7(N53S), but not CRT/E7, DNA vaccine elicits a human HLA-A2-restricted E7 specific T cell mediated immune response in HLA-A2 (AAD) transgenic mice by tetramer analysis

**Fig. 3:**
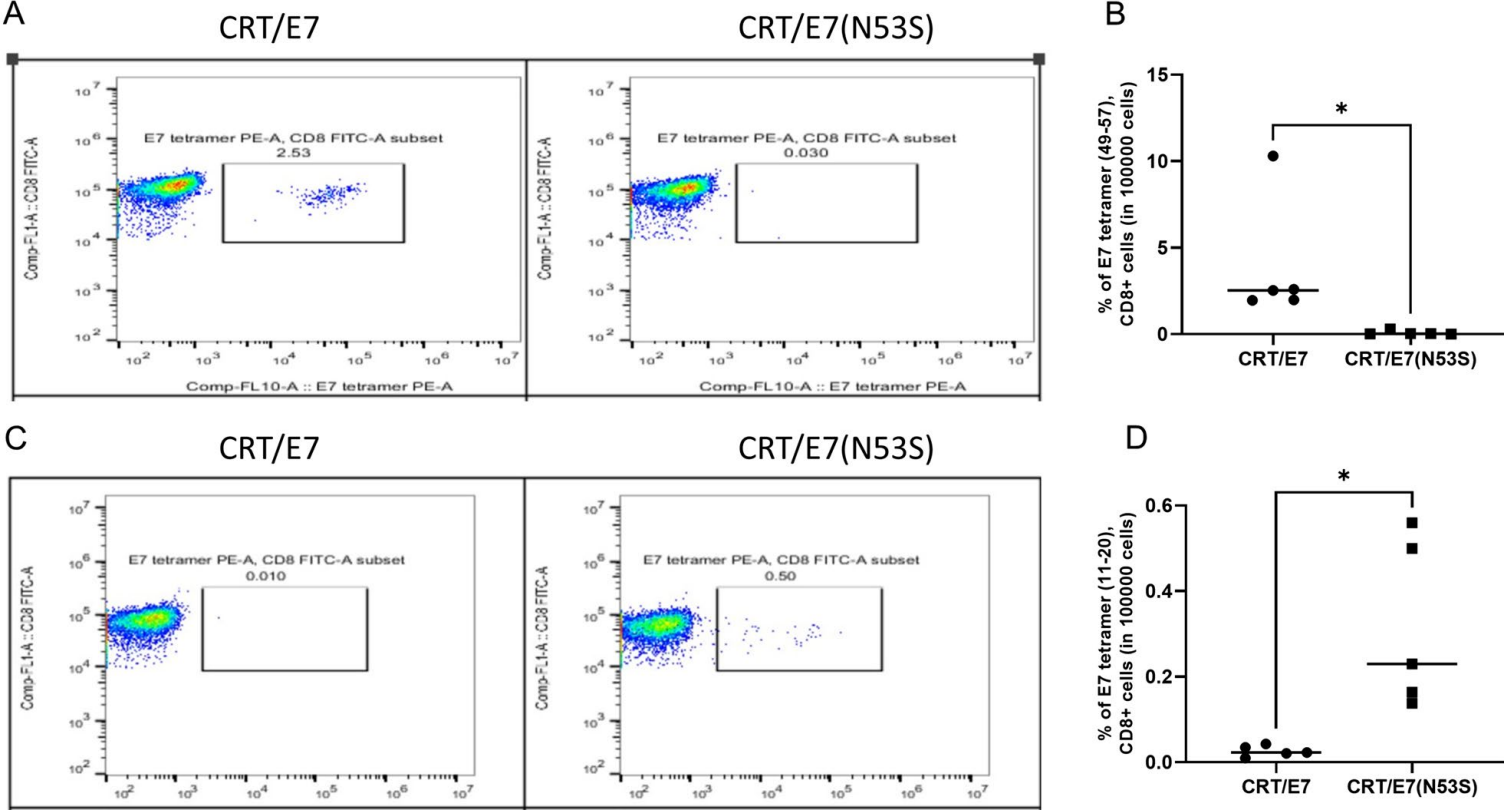
Comparison of HLA-A2 AAD transgenic mice vaccinated with CRT/E7 or CRT/E7(N53S) on their ability to generate murine H-2D^b^ restricted E7 peptide (aa49-57)-specific or HLA-A2 restricted E7 peptide (aa11-20)-specific CD8 + T cell immune response. Healthy HLA-A2 (AAD) transgenic mice (5 per group) were injected with either CRT/E7 or CRT/E7(N53S). The DNA vaccines were delivered to the shaved leg region of mice through intramuscular injection followed by electroporation (10 µg/plasmid, 30 µl/injection). Vaccines were injected twice a week for two consecutive weeks. One week after final vaccination, PBMCs from vaccinated mice were isolated and stained for CD8 + E7 peptide (aa11-20) loaded HLA-A2 tetramer + or E7 peptide (aa49-57) loaded H-2D^b^ tetramer + T cells and analyzed by flow cytometry analysis. **A** Representative flow cytometry analysis of PBMCs. The percentage of E7 peptide (aa49-57) loaded- H-2D^b^ tetramer positive T cells is shown in the number above the box. **B** Vertical scatter plot summarizing the data derived from E7 peptide (aa49-57)-loaded H-2D^b^ tetramer and CD8 + staining from mice vaccinated with CRT/E7 or CRT/E7(N53S) DNA. **C** Representative flow cytometry analysis of PBMCs. The percentage of E7 peptide (aa11-20) loaded- HLA-A2 tetramer positive T cells is shown in the number above the box. **D** Vertical scatter plot summarizing the data derived from E7 peptide (aa11-20)-loaded HLA-A2 tetramer and CD8 + staining from mice vaccinated with CRT/E7 or CRT/E7(N53S)

To further evaluate the HLA-A2-restricted E7-specific CD8^+^ T-cell immune response in HLA-A2 (AAD) transgenic mice vaccinated with CRT/E7 or CRT/E7(N53S) by peptide loaded MHC I tetramer analysis, we used PBMCs derived from HLA-A2 (AAD) transgenic mice vaccinated with CRT/E7 or CRT/E7(N53S). Two weeks after the final vaccination, PBMCs were collected from vaccinated mice and characterized by the murine E7 peptide (aa49-57) loaded H-2D^b^ tetramer or the human E7 peptide (aa11-20) loaded HLA-A2 tetramer. As shown in Fig. 3A and B, mice vaccinated with CRT/E7, but not CRT/E7(N53S), DNA generated the murine H-2D^b^ restricted E7 peptide (aa49-57)-specific T cell mediated immune responses. In comparison, mice vaccinated with CRT/E7(N53S), but not CRT/E7, DNA generated human HLA-A2 restricted E7 peptide (aa11-20)-specific T cell mediated immune responses (Fig. 3C and D). Our data from the tetramer analysis shows that the mutated E7 DNA vaccine leads to enhanced human HLA-A2 restricted E7 peptide (aa11-20)-specific T mediated immune responses, which is consistent with the data generated from the IFN-γ intracellular cytokine stain followed by flow cytometry analysis (Fig. 2).

### Injection of DNA constructs encoding HPV16-E6/E7, NRas^G12V^, sleeping beauty transposase 100, and luciferase followed by electroporation results in development of spontaneous HPV16E6E7-expressing oral tumor model in HLA-A2 (AAD) transgenic mice

**Fig. 4:**
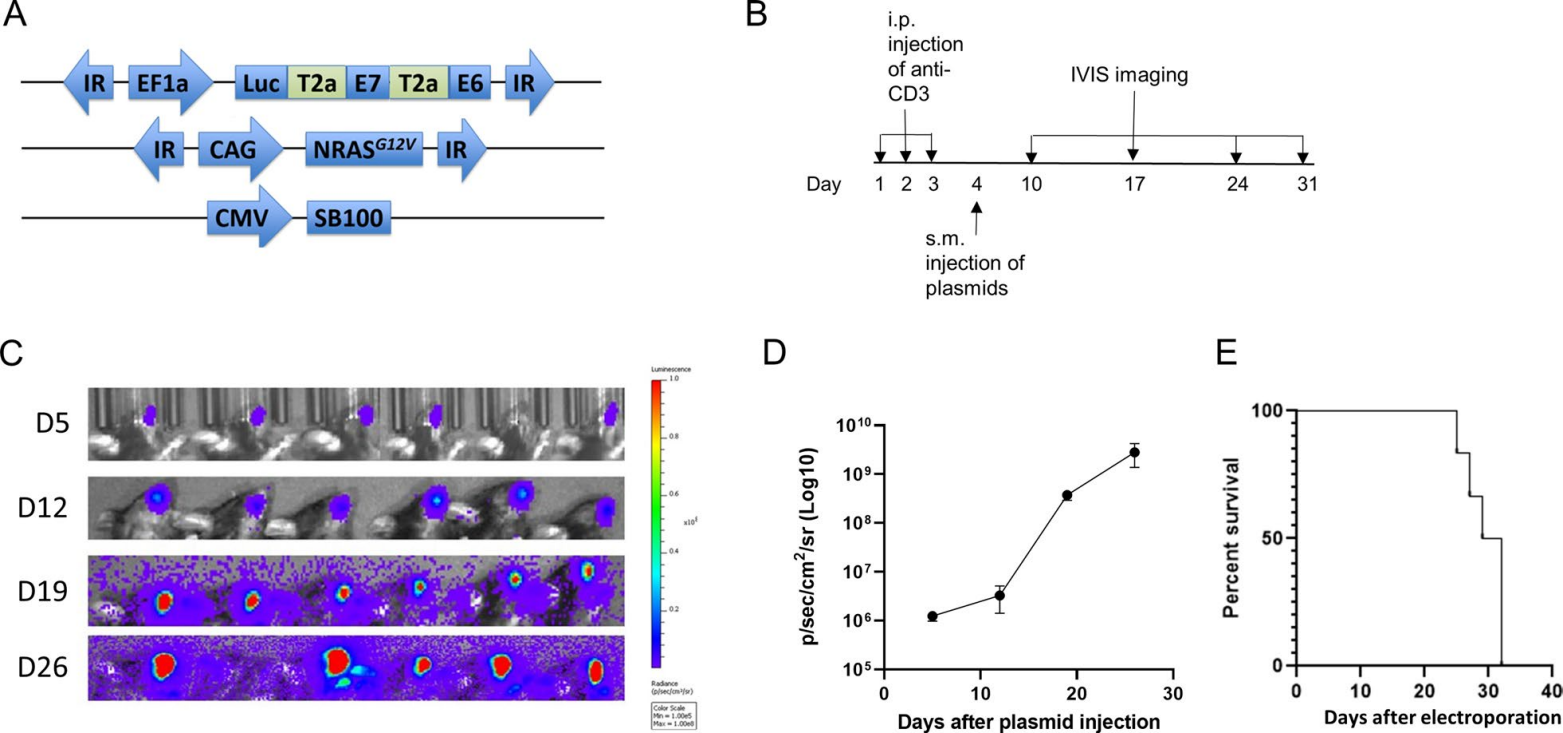
Characterization of a spontaneous HPV16-E6/E7-expressing oral tumor model in HLA-A2 (AAD) transgenic mice. **A** Schematic diagram of the four DNA constructs encoding HPV16 E6/E7, Ras, and SB100 DNA used for tumor model. **B** Schematic diagram of the experimental procedure. Six to 8 week old HLA-A2 (AAD) transgenic mice (6 per group) were injected with anti-CD3 monoclonal antibody via intraperitoneal (i.p.) injection three times (150 µg/mouse). On the fourth day, mice were injected submucosally (s.m.) with the DNA constructs encoding Ras, SB100, and HPV16E6/E7 (10 µg/plasmid, 30 µl/injection per mouse) into the oral area followed by electroporation. Mice were monitored for tumor growth via IVIS imaging every week for 4 weeks. **C **Bioluminescence imaging of tumor bearing mice. **D** Line graph summarizing the bioluminescence imaging of the tumor growth over time. **E** Kaplan–Meier survival curve of mice bearing HPV16-E6/E7-expressing oral tumor

To generate spontaneous oral tumors that continuously express HPV16-E6/E7, four DNA plasmids encoding HPV16-E6/E7, NRas^G12V^, sleeping beauty transposase 100 (SB100), and luciferase were generated (Fig. 4A). HLA-A2 (AAD) were initially depleted for CD3 T cells by intraperitoneal injection of anti-CD3 specific monoclonal antibodies daily for three consecutive days. The DNA constructs were subsequently submucosally injected into the oral area of mice, followed by electroporation on the third day (Fig. 4B). The tumor growth was followed by luminescence imaging. The luciferase enzymes encoded in the plasmids resulted in the generation of bioluminescence from viable, transfected cells that could be measured quantitatively using an IVIS imager. As shown in Fig. 4C and D we observed that all the mice receiving the injection of combination DNA resulted in increased luminescence intensity overtime. All of the mice developed palpable tumor (data not shown). The tumor growth eventually resulted in the death of 100% of the tumor bearing mice (Fig. 4E). Taken together, our data indicates that this combination of constructs encoding SB100, luciferase, HPV16-E6E7, and NRas^G12V^ oncogenes resulted in fast tumor formation and growth rate as measured by the luminescence intensity**.**

### Treatment with CRT/E7(N53S) DNA results in tumor control and prolonged survival in mice bearing spontaneous HPV16-E6E7-expressing oral tumors

**Fig. 5:**
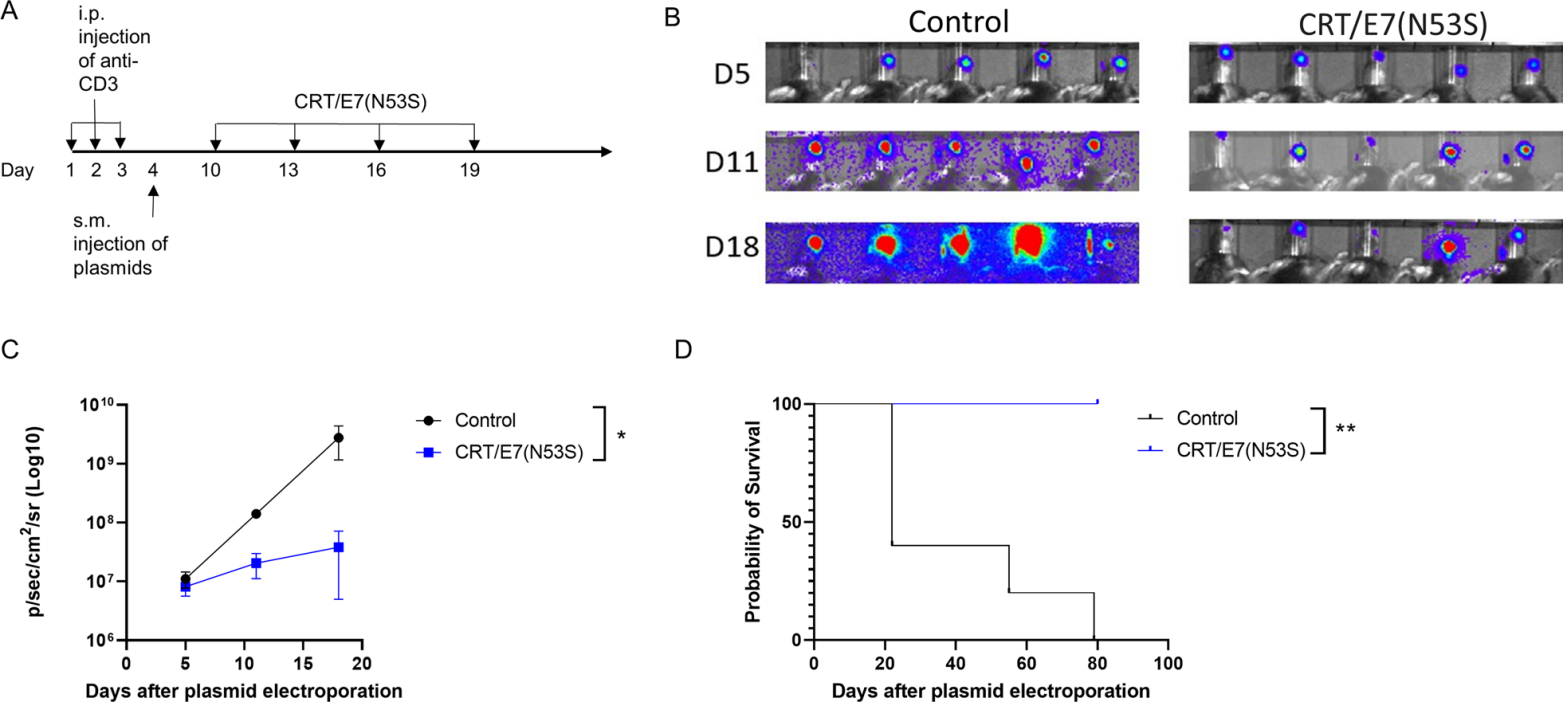
Characterization of therapeutic antitumor effect of CRT/E7(N53S) vaccination against the spontaneous HPV16-E6E7-expressing oral tumor model.** A** Schematic diagram of the experimental procedure. HPV16-E6E7-expressing oral tumors were induced in HLA-A2 (AAD) transgenic mice (5 per group) as described in Fig. 4. One week after final anti-CD3 injection, tumor bearing mice were then treated with either CRT/E7(N53S) DNA (10 µg/dose/mouse) or empty pcDNA vector (10 µg/dose/mouse) by i.m. injection in the hind leg, followed by electroporation. The mice continued to receive the same vaccination at 4-day intervals 3 additional times. Tumor growth was monitored on days 5, 11, and 18 after the final vaccination. **B** Bioluminescence imaging of tumor bearing mice treated with CRT/E7(N53S) DNA or empty vector. **C** Line graph summarizing the luminescent imaging of tumor bearing mice. **D** Kaplan–Meier survival curve of HPV16-E6E7-expressing oral tumor bearing mice who received CRT/E7(N53S) or empty vector

In order to determine if CRT/E7(N53S) can generate a therapeutic antitumor effect against the spontaneous HPV16-E6E7-expressing oral tumor, HLA-A2 (AAD) transgenic mice were injected with HPV16-E6E7-expressing oral tumor using HPV16-E6/E7, NRas^G12V^, SB100, and luciferase reporter constructs as described in Fig. 4 to create the tumor model. The tumor bearing mice were treated with either CRT/E7(N53S) or empty pcDNA3 vector (Fig. 5). The control group did not receive vaccination while the experimental groups were intramuscularly injected with 10 µg of CRT/E7(N53S) plasmids followed by electroporation twice a week for two consecutive weeks following plasmid injection. On day 5 post tumor introduction, control groups and vaccination groups had similar tumor growth signals. By day 11 and 18, the control group displayed more intense signals and tumor growth than the vaccinated group (Fig. 5A, B). By days 11 and 18, the tumors in the vaccinated group were no longer growing by visual observation, and the growth did not continue to increase. The luciferin activity was measured and quantified as photon counts and result is shown in Fig. 5C. This illustrates that the unvaccinated group experienced faster tumor growth compared to the vaccinated group. In addition, all five mice in the untreated group died within 80 days after plasmid injection, whereas all mice that received CRT/16E7 survived until the end of the experiments (post 100 days) (Fig. 5D).

### Tumor microenvironment analysis suggest that vaccination with CRT/E7(N53S) DNA results in enhanced effector CD8 + T cell response and reduces MDSCs

**Fig. 6:**
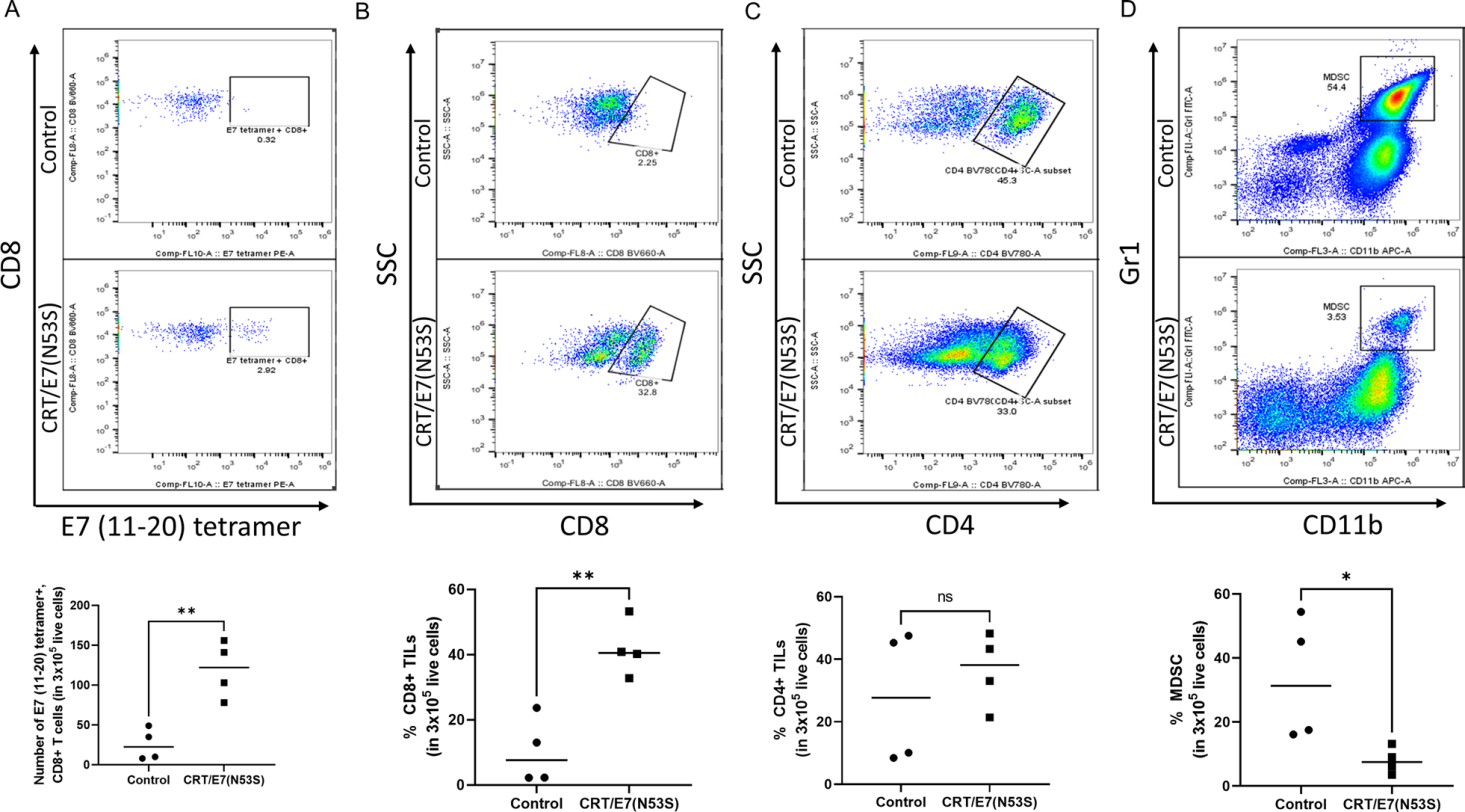
Characterization of the tumor microenvironment in spontaneous HPV16-E6E7-expressing oral tumor model in HLA-A2 (AAD) transgenic mice treated with CRT/E7(N53S) DNA vaccine. HPV16-E6E7-expressing oral tumors were induced in HLA-A2 (AAD) mice (4 per group) and treated as described in Fig. 5. One week following the final vaccination, the tumor was surgically extracted for analysis. **A** Determination of E7(aa11-20) peptide-specific CD8 + T cells using E7 (aa11-20) peptide loaded HLA-A2 tetramer staining followed by flow cytometry analysis. Upper panel is an example of flow cytometry. Bottom panel is vertical scatter plot summarizing the number of E7(aa11-20) peptide-specific CD8 + T cells in the tumor of mice vaccinated with CRT/E7(N53S) or empty vector. **B** Determination of CD8 + T TILs by flow cytometry analysis. Upper panel is an example of flow cytometry. Bottom panel is the vertical scatter plot summarizing the percent of CD8 + TILs. **C** Determination of CD4 + TILs followed by flow cytometry analysis. Upper panel is an example of flow cytometry. Bottom panel is the vertical scatter plot summarizing the percent of CD4 + TILs. **D** Determination of MDSCs by flow cytometry analysis. Upper panel is example of flow cytometry. Bottom panel is the vertical scatter plot summarizing the percent of MDSCs

We further characterized the tumor microenvironment in the tumor bearing HLA-A2 (AAD) transgenic mice vaccinated with either CRT/E7(N53S) DNA or empty DNA vector. Tumors from these vaccinated mice were explanted and the tumor infiltrating lymphocytes (TILs) were isolated for further characterization using flow cytometry analysis. As shown in Fig. 6A, there is a significant increase of E7(aa11-20) specific CD8 + T cells in the tumor from mice vaccinated with CRT/E7(N53S) compared to tumor from the mice vaccinated with empty vector. Furthermore, there is a significant increased percentage of CD8 + T cells among all the TILs in the tumor from mice vaccinated with CRT/E7(N53S) compared to the tumor from mice in the control group (Fig. 6B). In comparison, we did not observe a significant increase in percentage of CD4 + T cells in the CRT/E7(N53S) DNA vaccinated group (Fig. 6C). Furthermore, we also observed a significant reduction in the percentage of MDSCs in the tumor in the mice vaccinated with CRT/E7(N53S) DNA compared to the control group (Fig. 6D). Our data indicates that the vaccination with CRT/E7(N53S) was able to generate a favorable antitumor immune responses in the tumor microenvironment.

## Discussion

Here, we present the development of a pre-clinical HPV16 E6/E7-expressing oral tumor model in HLA-A2 (AAD) transgenic mice using oncogenic plasmids encoding sleeping beauty transposase, NRas^G12V^, and HPV16 E6/E7 oncogenes. We additionally observed that vaccination with mutated CRT/E7(N53S) DNA vaccine resulted in therapeutic antitumor effects and generated human HLA-A2 restricted HPV16 E7 specific CD8 + T cells rather than murine H-2D^b^ restricted HPV16 E7 specific CD8 + T cells.

Since the HLA-A2 (AAD) transgenic mice express both HLA-A2 (AAD) and murine H-2 D^b^ molecules, the E7 protein can potentially be presented through either human MHC class I molecules or murine MHC class I molecules. However, in the current studies, we observed that vaccination with CRT/E7 DNA vaccine only generated murine H-2D^b^ restricted E7 peptide (aa49-57)-specific CD8 + T cell mediated immune responses but not human HLA-A2 restricted E7 specific CD8 + T cell mediated immune responses (see Fig. 1). These results suggest that the presence of the immunodominant murine H-2D^b^ restricted E7 peptide (aa49-57) CTL epitope in the wild type E7 gene of the CRT/E7 DNA vaccine may suppress the presentation of the various human HLA-A2 restricted E7-specific CTL epitopes (including aa11-20, aa82-90 and aa86-93). We further confirm the phenomenon using the mutated E7 gene which changes one amino acid at the location of 53 in E7 gene (from N to S) to alter the murine H-2D^b^ restricted E7 peptide (aa49-57)-specific immunodominant CTL epitope. We found that HLA-A2 (AAD) transgenic mice vaccinated with CRT/E7(N53S) DNA vaccine generated only human HLA-A2 restricted E7-specific CD8 + T cell mediated immune responses but not murine H-2D^b^ restricted E7-specific CD8 + T cell mediated immune responses (see Fig. 2). Taken together, our data supports that the immunodominant murine H-2D^b^ restricted E7 peptide (aa 49–57) CTL epitope in the wild type E7 gene of the CRT/E7 DNA vaccine can suppress the presentation of the various human HLA-A2 restricted E7-specific CTL epitopes.

While vaccination of HLA-A2(AAD) transgenic mice with CRT/E7 DNA could not generate HLA-A2 restricted E7-specific CD8 + T cell mediated immune responses and vaccination with CRT/E7(N53S) DNA vaccine could not generate detectable murine H-2D^b^ restricted E7 specific CD8 + T cell mediated immune responses, vaccination of wild type E7-expressing spontaneous oral tumor bearing mice with CRT/E7(N53S) DNA vaccine in HLA-A2 (AAD) transgenic mice was capable of controlling tumor growth and results in prolonged survival for tumor bearing mice compared to empty vector (Fig. 5). Although CRT/E7(N53S) is able to control wild-type E7 expressing tumor in HLA-A2 (AAD) transgenic mice, the mutated E7 vaccine does not generate an immune response against HPV16 E7(aa49-57) epitope in CRT/E7(N53S) vaccinated HLA-A2 transgenic mice (Figs. 2 and 3). Taken together, these results suggests that the wild type E7 expressing tumor may also present HPV16 E7(aa11-20)-specific CTL epitope through HLA-A2 molecule in order to be controlled. Our data implies that the presence of HPV16 E7 (aa49-57) CTL epitope in wild type E7 -expressing tumor does not seem capable of completely suppressing the presentation of HPV16 E7(aa11-20) through HLA-A2 molecule in the tumor model as the tumor can be controlled by vaccination with CRT/E7(N53S) DNA vaccine.

It is still unclear why wild-type HPV16 E7 can still present human HLA-A2 restricted HPV16 E7(aa11-20) specific CTL epitope without being completely suppressed by murine H-2D^b^ restricted HPV16 E7(aa49-57)-specific CTL epitope in tumor bearing HLA-A2 (AAD) transgenic mice while the expressing of wild type E7 in CRT/E7 chimeric protein in vaccinated mice completely abolishes the presentation of murine H-2 D^b^ restricted CTL epitope (aa49-57). One possibility is that linkage of CRT to wild type E7 makes the presentation of murine H-2D^b^ restricted E7-specific CTL epitope (aa49-57) predominant to a greater degree than when the wild type E7 protein is not linked to CRT, such as in the tumor model. Alternatively, HPV16 E7 presentation could be different in tumor cells versus in immune cells. Further experimental research that determines the degree to which tumor cells can present HPV16 E7(aa11-20) via activation analysis would be required to better determine the reason behind this observed result.

Our HPV16E6E7-expressing oral tumor model recapitulates several important features that are seen clinically. However, there is one major drawback to this model. Most HPV-associated HN cancers present as carcinomas, whereas our model is more indicative of sarcoma (Additional file 1: Fig. S1A, B). Recently, we have identified that AKT and c-myc oncogenes can generate carcinoma morphology in a preclinical cervical SCC model [27]. Therefore, it would be of interest to improve upon the currently presented oral model by including oncogenic plasmids with AKT and c-myc in the transgenic HLA-A2 (AAD) mice.

## Conclusions

In all, we have successfully built a spontaneous HPV16 E6E7-expressing oral tumor model in HLA-A2 (AAD) transgenic mice and showed that CRT/E7(N53S) can elicit antitumor effects. In addition, we demonstrated that CRT/E7(N53S) can elicit human HLA-A2-restricted E7 specific T cell mediated immune responses (Fig. 2) and can induce an antitumor immune environment (Fig. 6). The model and mutations from this study can be applied in other preclinical studies to enhance translational potential of preclinical models and ensure human MHC I presentation is not out-competed by murine H-2D^b^ presentation.

## Supplementary Information

**Additional file 1: Figure S1.** Gross morphology and histological examination of HPV16-E6E7-expressing oral tumor model in HLA-A2 (AAD) transgenic mice.

## Data Availability

All data and materials are available from the corresponding authors upon written request.
