# Supplementary figures and images for: Control of Spontaneous HPV16 E6/E7 Expressing Oral Cancer in HLA-A2 (AAD) Transgenic Mice with Therapeutic HPV DNA Vaccine
Source: J Biomed Sci. 2021 Sep 13;28:63. doi: 10.1186/s12929-021-00759-x (PMC8436567; doi:10.1186/s12929-021-00759-x)

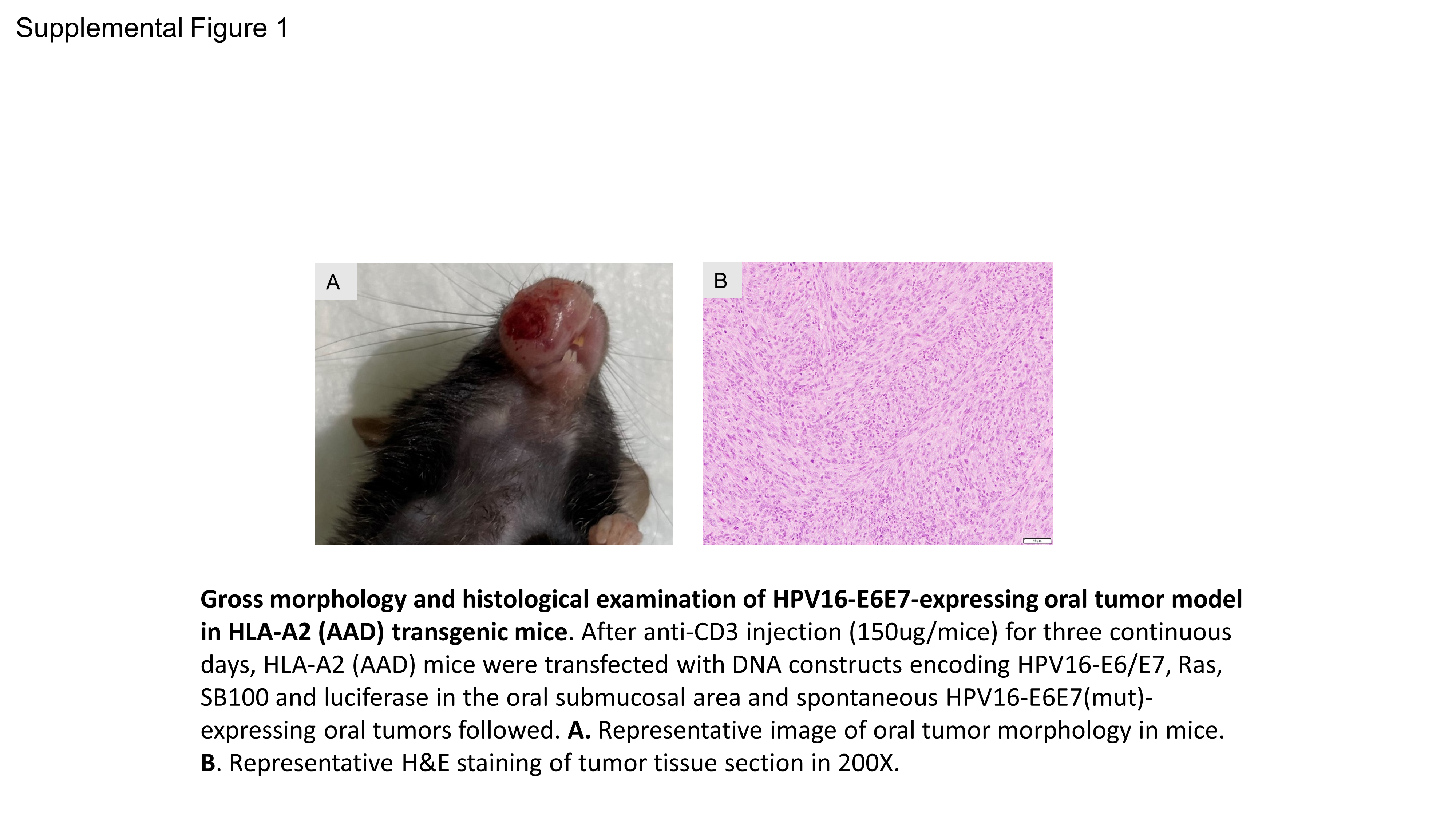

Supplement: Supplementary file 1 — Additional file 1: Figure S1. Gross morphology and histological examination of HPV16-E6E7-expressing oral tumor model in HLA-A2 (AAD) transgenic mice. [file 12929_2021_759_MOESM1_ESM.png]
